# Clinical factors associated with patterns of endocrine therapy adherence in premenopausal breast cancer patients

**DOI:** 10.1186/s13058-024-01819-4

**Published:** 2024-04-08

**Authors:** Kirsten M. Woolpert, Julie A. Schmidt, Thomas P. Ahern, Cathrine F. Hjorth, Dóra K. Farkas, Bent Ejlertsen, Lindsay J. Collin, Timothy L. Lash, Deirdre P. Cronin-Fenton

**Affiliations:** 1 Department of Clinical Epidemiology, Department of Clinical Medicine, Aarhus University and Aarhus University Hospital, Aarhus, Denmark; 2 Department of Surgery, The Robert Larner, M.D. College of Medicine at the University of Vermont, Burlington, VT USA; 3 On behalf of the Danish Breast Cancer Group, Rigshospitalet, Copenhagen, Denmark; 4 Department of Oncology, Department of Clinical Medicine, University of Copenhagen and Rigshospitalet, Copenhagen, Denmark; 5 Department of Population Health Sciences, Huntsman Cancer Institute, University of Utah, Salt Lake City, UT USA; 6 Department of Epidemiology, Rollins School of Public Health, Emory University, Atlanta, GA USA

**Keywords:** Adherence, Adjuvant endocrine therapy, Premenopausal breast cancer, Clinical characteristics, Comorbidities, Chronic medication use

## Abstract

**Introduction:**

Patients with hormone receptor positive breast cancer are recommended at least five years of adjuvant endocrine therapy, but adherence to this treatment is often suboptimal. We investigated longitudinal trends in adjuvant endocrine therapy (AET) adherence among premenopausal breast cancer patients and identified clinical characteristics, including baseline comorbidities and non-cancer chronic medication use, associated with AET adherence.

**Methods:**

We included stage I–III premenopausal breast cancer patients diagnosed during 2002–2011 and registered in the Danish Breast Cancer Group clinical database who initiated AET. We used group-based trajectory modeling to describe AET adherence patterns. We also linked patients to Danish population-based registries and fit multinomial logistic models to compute odds ratios (ORs) and 95% confidence intervals (95% CIs) associating clinical characteristics with AET adherence patterns.

**Results:**

We identified three adherence patterns among 4,353 women—high adherers (57%), slow decliners (36%), and rapid decliners (6.9%). Women with stage I disease (vs. stage II; OR: 1.9, 95% CI 1.5, 2.5), without chemotherapy (vs. chemotherapy; OR: 4.3, 95% CI 3.0, 6.1), with prevalent comorbid disease (Charlson Comorbidity Index score ≥ 1 vs. 0; OR: 1.6, 95% CI 1.1, 2.3), and with a history of chronic non-cancer medication use (vs. none; OR: 1.3, 95% CI 1.0, 1.8) were more likely to be rapid decliners compared with high adherers.

**Conclusions:**

Women with stage I cancer, no chemotherapy, higher comorbidity burden, and history of chronic non-cancer medication use were less likely to adhere to AET. Taking steps to promote adherence in these groups of women may reduce their risk of recurrence.

**Supplementary Information:**

The online version contains supplementary material available at 10.1186/s13058-024-01819-4.

## Background

Adjuvant endocrine therapy (AET) roughly halves the risk of recurrence among the two-thirds of premenopausal breast cancer patients whose tumors over-express the estrogen receptor (i.e., estrogen receptor positive [ER+]) [1, 2]. Breast cancer patients with ER+ tumors are recommended to take AET for a minimum of five years [3]. The standard AET for premenopausal women with breast cancer is tamoxifen (TAM) in patients at low or intermediate recurrence risk, and TAM or aromatase inhibitors (AI) plus ovarian function suppression in women with high recurrence risk [3, 4, 5]. Women who transition to menopause, who experience severe side effects, or who are treated with ovarian function suppression are recommended to switch to an AI [3, 3, 6, 7, 8]. Newer treatments, such as cyclin-dependent kinase inhibitors, are often taken concomitantly with AET in high-risk patients [9]. Side effects such as depression, nausea, and hot flashes may also impact adherence to AET [10, 11]. In a study examining AET adherence in the Danish Breast Cancer Group (DBCG) clinical database, we found that approximately 22% of premenopausal women discontinued AET during the intended treatment period. Those who discontinued had a higher rate of recurrence compared with those who completed treatment (hazard ratio [HR] = 1.67, 95% CI 1.25, 2.14) [12]. As patients with lower adherence to AET have a poorer prognosis, it is important to identify predictors of poor adherence. Such predictors could inform groups of women that would benefit most from adherence-enhancing interventions, which would minimize recurrence risk and prolong survival.

Previous studies have found that various factors—including patient characteristics, the type of healthcare system, and socioeconomic position—influence AET adherence [13]. These studies investigated the implementation of AET, as measured by the Medication Possession Ratio or Proportion of Days Covered (PDC) dichotomized at 80%, or focused on non-persistence, or gaps in prescription redemption [13, 14]. Although a summary statistic for adherence can simplify analyses, one composite metric cannot fully capture the dynamics of the complex behavior surrounding medication adherence. Few studies have considered how clinical factors influence changing patterns of AET adherence over the recommended five years of treatment [15, 16, 17].

Group-based trajectory models capture longitudinal patterns by enumerating patterns that represent the evolution of medication adherence over time [18, 19, 20, 21]. In a US study of adherence in the 12 months following AET initiation, trajectory group assignment was associated with mortality; for example, those with a ‘quick decline’ had a higher risk of death compared with those with high adherence (HR: 1.41, 95% CI 1.09, 1.72]) [22]. Studies investigating factors influencing AET adherence with a group-based trajectory approach found that no chemotherapy treatment [15], and younger or older age (vs. middle age) [16, 17] are associated with declining AET adherence. However, these studies were prone to selection bias [15] and lacked information on clinical characteristics [16, 17]. The structure of AET adherence patterns among a population-based cohort of exclusively premenopausal women remains unknown.

Our aim was to evaluate patterns of adherence to AET in a cohort of breast cancer patients who were premenopausal at diagnosis. We further aimed to examine adherence to AET during follow-up according to patient, tumor, and treatment characteristics, as well as baseline comorbidities and prior non-cancer chronic medication use.

## Methods

### Source population

The Predictors of Breast Cancer Recurrence (ProBe CaRe) cohort includes 5,959 premenopausal women diagnosed with stage I–III primary breast cancer between 2002 and 2011 in Denmark and registered in the DBCG [23]. For the current study, we restricted the cohort to women classified as ER+ (n = 4,600). In line with the national guideline change of the definition of ER+ in 2010, patients diagnosed before the guideline change had a cut-off of 10% ER positivity, and those diagnosed after had a cut-off of 1% ER positivity [24]. We excluded women with less than one year of follow-up after initiation of AET (n = 104), as the group-based trajectory models were unstable in women with less than two follow-up visits. We also excluded women who were 55 years or older at diagnosis or who initiated treatment with an AI (n = 118), and women who initiated AET before breast cancer diagnosis, after a recurrence, or after diagnosis of another malignancy (n = 25).

### Analytic variables

#### Clinical factors, comorbidities, and prior medications

The DBCG routinely collects information on demographic, clinical, and treatment characteristics after breast cancer diagnosis [25]. The clinical factors we explored in relation to group trajectory assignment included characteristics of the tumor (stage, tumor size, lymph node status, histologic grade, and human epidermal growth factor receptor 2 [HER2] status), surgery and radiation type, chemotherapy treatment, and age at cancer diagnosis. We also investigated prevalent comorbidities in all years before breast cancer diagnosis by calculating the Charlson Comorbidity Index (CCI) score for every individual in the cohort from available diagnoses registered in the Danish National Patient Registry, which dates back to 1977 and covers all Danish hospitals [42, 43].

**Fig. 1:**
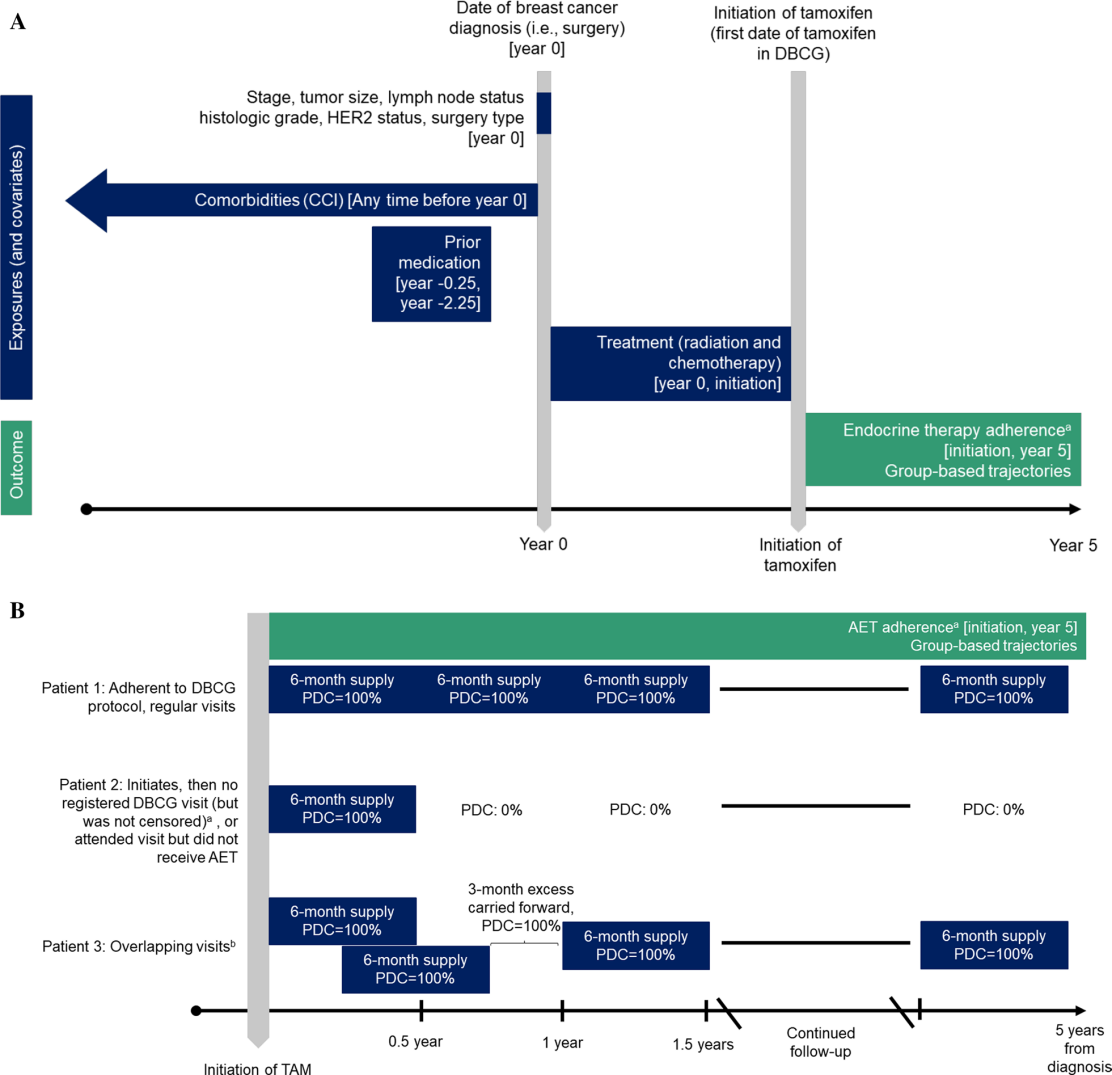
**A** Study design used to describe associations among patient/tumor/treatment characteristics, baseline comorbidities, and prior non-cancer chronic medication use and patterns of adherence to adjuvant endocrine therapy (AET). **B** Hypothetical patient scenarios for measuring patterns of adjuvant endocrine therapy adherence (AET) in premenopausal breast cancer patients during five years of follow-up. *Abbreviations* TAM, tamoxifen; DBCG, Danish Breast Cancer Group; AET, adjuvant endocrine therapy; CCI, Charlson Comorbidity Index; PDC, proportion of days covered. ^a^Patients censored at time of recurrence, death, second primary cancer, emigration, or five years after breast cancer diagnosis. ^b^A maximum of 182 days (or 6-month) dosage was allowed to be carried forward to the next interval. ^c^Figures developed using Schneeweiss et al. template diagrams [27].

To investigate prior medication use, we linked our cohort to the Danish National Prescription Registry, which has recorded information on all filled prescriptions in Denmark since 1995 [26]. We defined the two-year period for prior medication use spanning 3–27 months before diagnosis to ensure drugs relating to breast cancer diagnosis were excluded. Any medication use was defined as at least two redeemed prescriptions for the same drug, requiring that the woman had the same Anatomical Therapeutic Chemical (ATC) classification code at least twice in the two-year window. We also investigated the most frequently prescribed medications in the cohort. These included psychoanaleptics, psycholeptics, thyroid medications, systemic hormonal contraceptives, analgesics, diuretics or antihypertensives, and/or obstructive airway or systemic antihistamines (Additional file 1: Table S1). Women using these selected medications also were defined as users if they had at least two redeemed prescriptions within the same category during the two-year window. In sensitivity analyses, we investigated medication use from three years to one year before diagnosis in the same way as described above. Exposure windows and study design aspects are depicted in Fig. 1A.

### Adherence to adjuvant endocrine therapy

#### Supply diaries

For the diagnostic period of our study cohort, DBCG guidelines recommended that women with breast cancer in Denmark undergo semi-annual examinations at their treating hospital for the first five years following diagnosis [25]. Women received free-of-charge AET directly from their treating physician at these follow-up visits, and not at pharmacies like in other healthcare settings. Women were also offered AET by mail between visits, if needed. DBCG logs information on the date of the visit, whether the patient was still taking AET, and the type of therapy prescribed (TAM or AI). If a patient attended their follow-up visit and reported continued AET use, it was assumed that they received a 6-month supply of AET (Fig. 1B, patient scenario 1). If a patient did not attend follow-up visits but still resided in Denmark, or attended their follow-up visit and reported no AET use, they were considered non-adherent (Fig. 1B, patient scenario 2). Using this information, we created a supply diary for each patient. Each supply diary began on the date of the first AET registration (i.e., index date, or initiation) and ended five years after the diagnosis date. If a woman had registered AET before the end of coverage from her previous period, we assumed that she was still adherent to her available medication for the subsequent period, allowing for a maximum of six months of dosage in the supply diaries to be carried forward to the next six-month interval (Fig. 1B, patient scenario 3). Patients were censored at time of recurrence, death, second primary cancer, emigration, last day on DBCG protocol, or five years from breast cancer diagnosis. Registered AI treatment after initiation with TAM was still considered adherence and was thus included in the time-varying PDC measurements.

#### Group-based trajectory modeling

To describe longitudinal patterns for the adherence trajectories, we defined the PDC for each woman within each six-month period. The information from the supply diaries was used for the PDC numerator (i.e., the total number of days that a patient had medication on hand). The denominator for each PDC metric was six months, or time from the start of the six-month period until supply diary censoring. We then used longitudinal PDCs to model adherence under a censored normal distribution using the “PROC TRAJ” SAS package [28]. This package incorporates the time-varying PDC variables that describe adherence within each six-month interval, the time scale, the number of desired groups (varying from two to seven), and the degree of the polynomial function used to model adherence over time (varying from zeroth to second order). The package then assigns each patient to the group with the highest predicted probability of membership.

We used a three-step approach for selecting the final group-based trajectory model for further analysis. In step one, we started with quadratic (second order) polynomials, varying the number of groups from two to seven [29, 30]. In step two, we determined functional forms of each group in the selected model. Finally, in post-selection assessment of the selected model, we investigated the probabilities of group membership and used spaghetti plots to visually inspect homogeneity in adherence patterns within the groups [30, 31]. Detailed model selection methodology is provided in the Additional file 1.

### Covariates

To estimate the associations of patient, tumor, and treatment factors with AET adherence trajectories, we treated each factor as an individual exposure and used Directed Acyclic Graphs (DAGs) to select a customized set of adjustment variables (Additional file 1: Fig. S2). Possible covariates included age, stage, chemotherapy, surgery and radiation, histological grade, HER2 status, CCI score, household income in the year before diagnosis (from the Danish Income Statistics Registry) [32], education level (from the Population’s Education Registry) [33], and cohabitation status (from the Danish Civil Registration System) [34].

### Statistical analysis

We calculated descriptive statistics for clinical characteristics stratified by AET adherence group. In the case of missing stage, tumor size, and lymph node status, we performed multiple imputation using available patient and tumor characteristics. We used the ‘mice’ package [35] in R version 4.0 (Vienna, Austria) to impute missing data 50 times, which were aggregated by calculating the mean value across all 50 datasets. This impacted less than 1% of the study population for stage, tumor size, and lymph node status. We fit multinomial logistic regression models to estimate odds ratios (ORs) and 95% confidence intervals (CIs) associating different clinical factors with group-based trajectory assignment of AET adherence. The group with highest adherence was used as the outcome reference group. Analyses were conducted using SAS version 9.4 (SAS Institute, Cary, NC) and R version 4.0 (Vienna, Austria).

This study was approved by Central Denmark’s Regional Ethics Committee (journal number 1-10-72-22-13), the Danish Breast Cancer Group, the Danish Data Protection Agency (Aarhus University number 2016-051-000001, #458), and adhered to the General Data Protection Regulation. The data in this study was compiled and analyzed within the secure servers of Statistics Denmark in accordance with Danish privacy laws.

## Results

Our study population consisted of 4,353 ProBe CaRe participants with ER+ tumors diagnosed 2002–2011 (Additional file 1: Fig. S1). The median time from diagnosis to initiation of AET was 6.3 months (interquartile range [IQR]: 5.5–8 months). Characteristics of the final cohort are shown in Table 1. In the first step of model selection, we found that three groups sufficed to describe the patterns of adherence in our cohort. In the second step of model selection, we chose a model that described high adherers with a constant polynomial, and two groups with distinct declining adherence patterns, each modeled with second-order polynomials. Further model selection results are provided in the Additional file 1: Tables S2, S3, Figs. S2, S3.

**Table 1:** Characteristics of 4,353 premenopausal breast cancer patients by patterns of endocrine therapy adherence during five years following diagnosis

	High adherers, n (%)	Slow decliners, n (%)	Rapid decliners, n (%)
Total	2,465 (57)	1,587 (36)	301 (6.9)
Age at diagnosis			
< 40	359 (15)	261 (16)	54 (18)
40–49	1,548 (63)	975 (61)	173 (57)
50–55	558 (23)	351 (22)	74 (25)
Charlson Comorbidity Index score			
None	2,234 (91)	1,382 (87)	259 (86)
One or higher	231 (9.4)	205 (13)	42 (14)
Stage at diagnosis^a^			
Stage I	549 (22)	465 (29)	106 (35)
Stage II	1,398 (57)	837 (53)	147 (49)
Stage III	518 (21)	285 (18)	48 (16)
Positive lymph nodes^a^			
None	832 (34)	637 (40)	147 (49)
One	658 (27)	391 (25)	60 (20)
Two or more	975 (40)	559 (35)	94 (31)
Histological grade^a^			
I	500 (20)	343 (22)	68 (23)
II	1,289 (52)	819 (52)	163 (54)
III	524 (21)	310 (20)	56 (19)
Unknown/not graded	135 (5.5)	101 (6.4)	13 (4.3)
Tumor size^a, b^			
< 2 cm	1,354 (55)	969 (61)	189 (63)
> 2-5 cm	1,019 (41)	< 580	< 115
> 5 cm	92 (3.7)	< 55	< 5
HER2 status			
HER2 negative	1,517 (62)	1,030 (65)	189 (63)
HER2 positive	301 (12)	227 (14)	51 (17)
Unknown/not measured	647 (26)	330 (21)	61 (20)
Treated with chemotherapy			
Yes	2,354 (96)	1,362 (86)	247 (82)
No	111 (4.5)	225 (14)	54 (18)
Surgery type and radiotherapy			
Mastectomy without radiotherapy	364 (15)	215 (14)	49 (16)
Mastectomy and radiotherapy	788 (32)	433 (27)	79 (26)
Lumpectomy	1,313 (53)	939 (59)	173 (57)

^a^Missing values were multiply imputed across 50 repeat datasets, and the average was rounded and recorded

^b^Due to Danish data privacy laws, small cells and any cells that would allow back-calculation are rounded

**Fig. 2:**
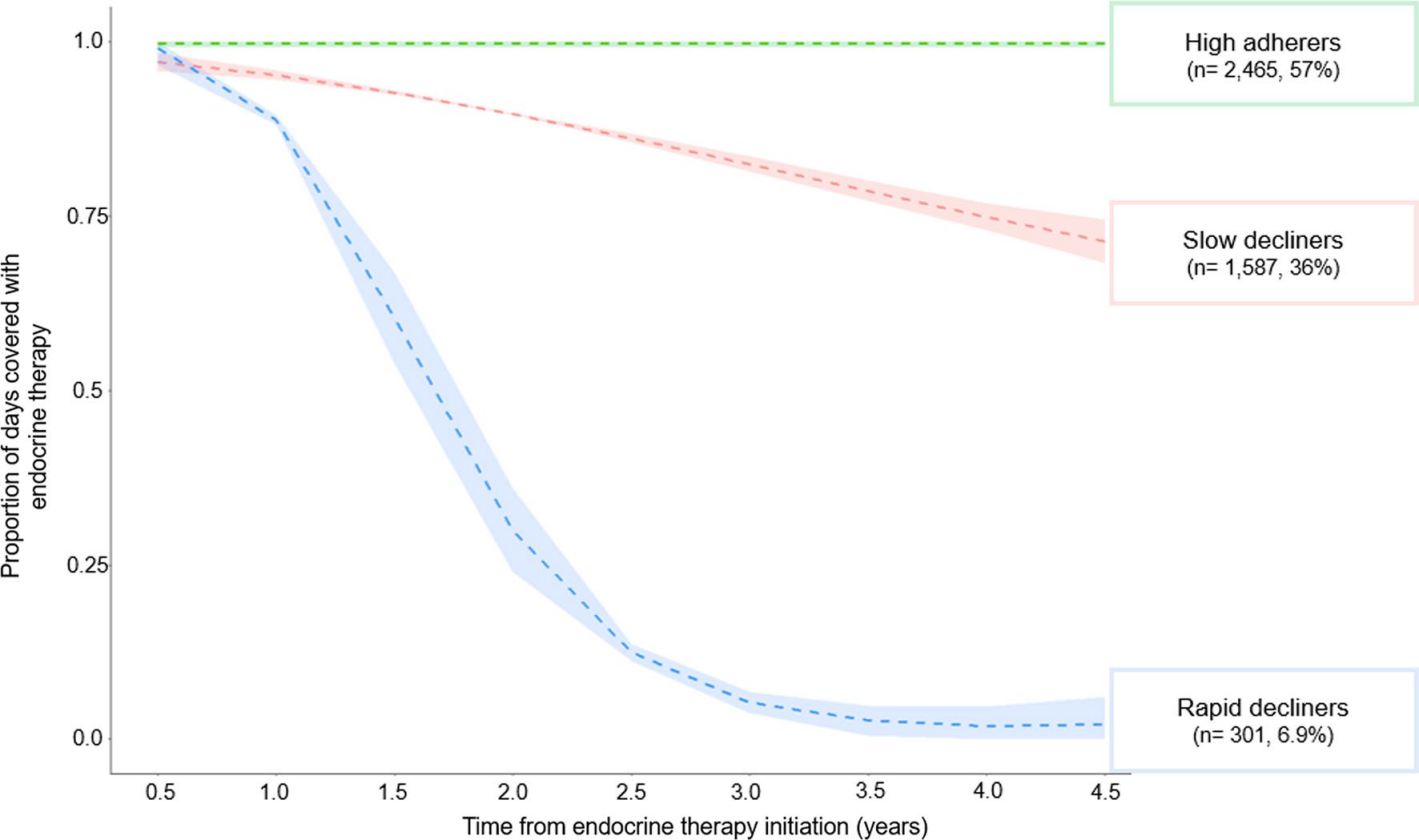
Group-based trajectories of endocrine therapy adherence in a cohort of 4,353 premenopausal women diagnosed with breast cancer in Denmark, diagnosed 2002–2011. ^a^Dotted lines represent the modeled estimate of the metric ‘proportion of days covered’ over time. The shaded area represents the 95% confidence interval around the model estimates

**Fig. 3:**
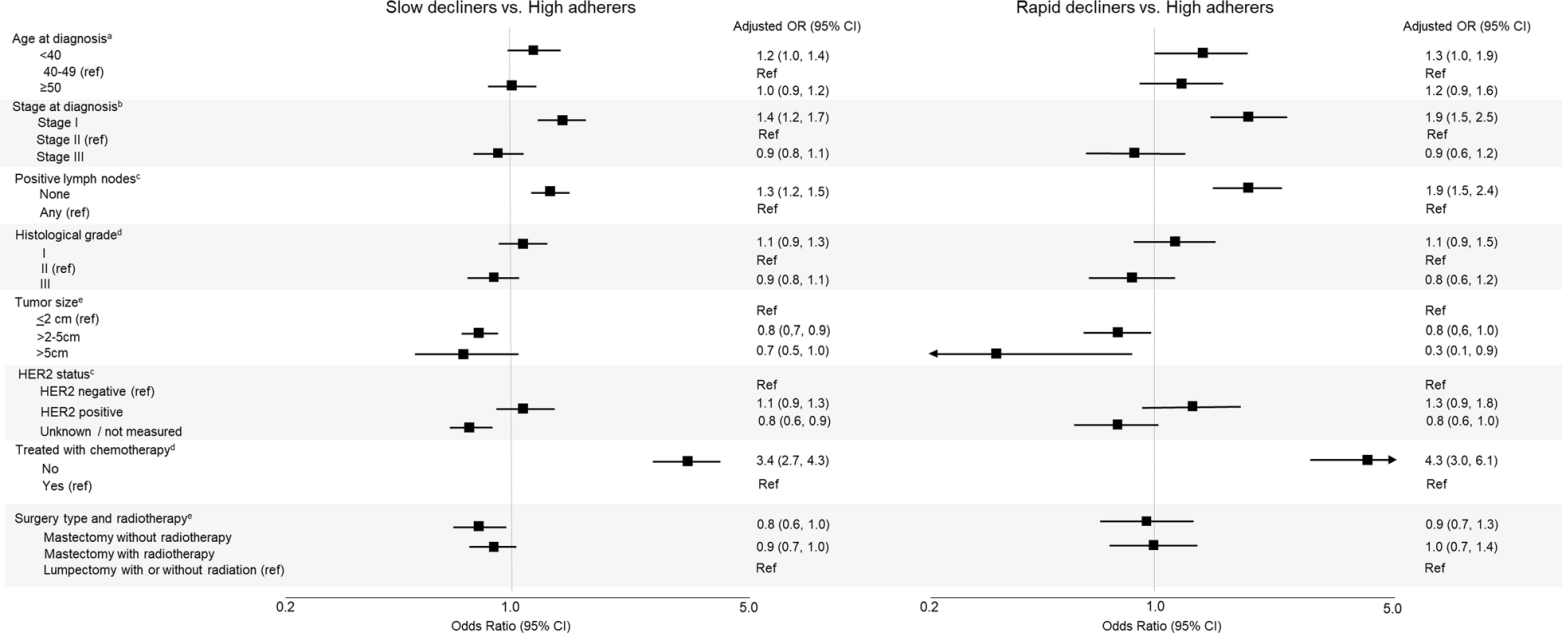
Associations of patient, tumor, and treatment characteristics with adherence to adjuvant endocrine therapy among 4,353 Danish premenopausal breast cancer patients diagnosed 2002–2011. Exposure reference groups were set to groups with the highest number of cohort members. *Abbreviations* OR, odds ratio; CI, confidence interval. Each model’s adjustment factors were directed by Directed Acyclic Graphs (Additional file 1: S2). ^a^Associations not adjusted for any covariates. ^b^Associations adjusted for age, Charlson Comorbidity Index score, household income, and cohabitation status. ^c^Associations adjusted for age. ^d^Associations adjusted for age, Charlson Comorbidity Index score, and cancer stage at diagnosis. ^e^Associations adjusted for age, Charlson Comorbidity Index score, cancer stage at diagnosis, and cohabitation status

In our final selected model, three patterns of adherence were identified— high adherers (n = 2,465, 57%), slow decliners (n = 1,587, 36%), and rapid decliners (n = 301, 6.9%) (Fig. 2). Women with stage I disease had higher odds of being slow decliners (OR: 1.4, 95% CI 1.2, 1.7) and rapid decliners (OR: 1.9, 95% CI 1.5, 2.5), compared with women with stage II disease. Other indicators of better prognosis were also related to higher odds of declining adherence (Fig. 3); for example, women with no positive lymph nodes at surgery were more likely to be slow decliners (OR: 1.3, 95% CI 1.2, 1.5) and rapid decliners (OR: 1.9, 95% CI 1.5, 2.4), compared with women with any positive nodes. The receipt of other cancer treatments also appeared to relate to patterns of AET adherence. Women who were not treated with chemotherapy were more likely to have declining adherence compared with women who received chemotherapy (slow decliners: OR: 3.4, 95% CI 2.7, 4.3; rapid decliners: OR: 4.3, 95% CI 3.0, 6.1).

**Fig. 4:**
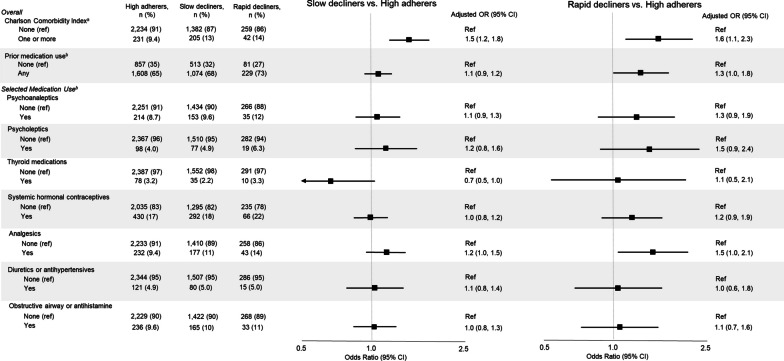
Associations of baseline comorbidities and prior non-cancer chronic medication use with adherence to adjuvant endocrine therapy 4,353 Danish premenopausal breast cancer patients diagnosed 2002–2011. Exposure reference groups were set to groups with the highest number of cohort members, except for estimates comparing any versus no prior medication use. The ‘None’ category was set as the reference a priori. Abbreviations: OR, odds ratio; CI, confidence interval. Each model’s adjustment factors were directed by Directed Acyclic Graphs (Additional file 1: S2). ^a^Associations between Charlson Comorbidity Index score and adherence adjusted for age and education level. ^b^Associations between prior medication use (and all selected medications) and adherence adjusted for age, Charlson Comorbidity Index score, household income, cohabitation status, and education level

We also found that women with comorbidity (i.e., CCI score ≥ 1) were more likely to be slow (OR: 1.5, 95% CI 1.2, 1.8) or rapid decliners (OR: 1.6, 95% CI 1.1, 2.3), compared with women with no comorbidities (Fig. 4). Some medication groups also appeared to increase the odds of declining adherence, including psychoanaleptics, psycholeptics, and analgesics (Fig. 4). For example, women who had at least two redeemed prescriptions for analgesics in a 2-year period before diagnosis had an increased odds of being rapid decliners compared with women without analgesic use (OR: 1.5, 95% CI 1.0, 2.1). Use of diuretics and antihypertensives did not appear to influence the odds of declining adherence (slow decliners OR: 1.1, 95% CI 0.8, 1.4; rapid decliners: OR: 1.0, 95% CI 0.6, 1.8). We observed similar null findings among women with fills of thyroid medications, systemic hormonal contraceptives, and obstructive airway/systemic antihistamines. Results from our sensitivity analysis, in which we changed the definition of prior medication use to a period further from the time of breast cancer diagnosis, did not differ meaningfully from the main analyses (Additional file 1: Fig. S5).

## Discussion

In this cohort of premenopausal women with early breast cancer, we found that patients with less advanced cancer (as measured by cancer stage, tumor size, and lymph node status) had poorer adherence to AET over follow-up. This association was also reflected in our findings related to cancer treatment, in which women who experienced a less rigorous treatment regimen (i.e., no chemotherapy) were less likely to remain adherent to AET, even after accounting for less advanced disease. Women with a higher comorbidity burden and those who regularly took certain medications before their cancer diagnosis had an increased risk of poorer adherence to AET. Specific medications that appeared to increase the risk of declining adherence included psycholeptics, psychoanaleptics, and analgesics. In a recent systematic review of AET adherence interventions, some approaches, such as psychosocial and reminder interventions, had some promise in promoting adherence [36]. However, the authors concluded that more powerful approaches would be needed to improve efficacy [36]. Our study provides further evidence of the subgroups of women who may benefit the most from these types of interventions. These subgroups of patients at risk for poorer adherence have not previously been identified in a cohort of strictly premenopausal women.

A recent study of young breast cancer patients suggested that women with less advanced cancer were less prone to have a “fear of recurrence.” [37] Such fear may motivate adherence to AET in some patients, and thus may partly explain our finding of lowest adherence in patients with the lowest expected risk of disease recurrence. Still, our previous research in patients with early breast cancer suggested that even women with earlier stage tumors have a notable risk of long-term recurrence. We observed a cumulative incidence of recurrence during 10 to 25 years of 12.7% (95% CI 11.9%, 13.5%) among node-negative patients with stage I tumors (T1N0) [38]. Stage I patients were also identified as having poorer adherence in the current study. It is possible that poor adherence could partly explain this previous finding of an unexpectedly high late recurrence risk among patients presumed to have the best prognosis. This may provide an opportunity to target specific subgroups at risk of discontinuing treatment prematurely and thereby experiencing a preventable recurrence.

Women who took psycholeptics, psychoanaleptics, or analgesics had increased odds of poorer AET adherence. These medications are used to treat depression, anxiety, insomnia, and chronic pain, which may highlight that women with these disorders are at high risk of poor adherence behavior. One study reported similar findings, where women with unipolar depression, anxiety, non-schizophrenia psychosis, and dementia were less likely to initiate and adhere to AET in the first year after diagnosis [39]. This study also found that women with a history of substance use disorder had 2.3% lower adherence to AET over 5 years (95% CI − 3.8%, − 0.9%) [39]. Importantly, the ProBe CaRe cohort is young and may be healthier than other cohorts of breast cancer patients, with a low number of non-cancer related prescriptions and comorbid diseases. Although our findings are consistent with similar studies, we did have poor precision for many of the estimates regarding non-cancer chronic medications. Due to this low statistical power, we were unable to investigate the specific comorbidities that may be driving these associations with AET adherence. Additionally, we were unable to look at concomitant chronic medication use and incident comorbidities. Given that side effects are among the major determinants of poor AET adherence, this study can only provide a partial characterization of AET adherence predictors.

These clinical factors that influence adherence to endocrine therapy have not been previously researched in the Danish setting. Of note, adherence to AET was relatively high in this cohort compared with breast cancer patients from other populations, with more than half of patients maintaining the highest level of adherence (57%). In a systematic review of 26 articles exploring the factors influencing adherence to AET, adherence up to five years of AET ranged from 33.3 to 88.6% [13]. A possible explanation for the high adherence in the ProBe CaRe cohort could relate to the Danish public welfare system, which provides society-wide healthcare through tax-financed services [40]. Studies have found that treatment adherence improves when healthcare copayments are reduced or eliminated [41, 44, 45]. Provision of AET in Denmark is at no cost to the patients, which could provide one explanation for differences in AET adherence between populations. Our findings of generally high AET adherence may be relevant to other countries with similar healthcare settings and systems. Additionally, patients in Denmark receive their AET directly from their treating physician, which may improve trust and reduce the burden of pharmacy visits. The possibility of higher nonadherence combined with the high risk of breast cancer recurrence in nonadherent patients sheds light on the importance for similar studies to be conducted in other populations.

As with any adherence study that relies on registry-based data, we do not know whether patients were taking the drug as prescribed. Also, the exact amount of AET received by a patient is not known, which may lead to some misclassification of adherence status. However, on average, women receive enough AET to last until at least the subsequent follow-up visit. Our assumption that a woman can carry-over up to six months of excess dose was a conservative approach and would likely attenuate effect estimates. There is also the possibility of misclassification of adherence status induced by the group-based trajectory modeling. Though the probabilities of membership were high, some individual-level patterns of adherence may not be fully represented by their final selected group pattern. Additionally, our exclusion of women with short follow-up after initiation, which was most often due to an early recurrence in the year following the start of AET, is also a limitation. We made this exclusion because those censored after only one follow-up visit introduced instability into our group-based trajectory modeling. With only one or two follow-up visits, these women did not have enough time to establish themselves as adherers or decliners. As this exclusion influenced only a small proportion of our overall cohort (2.4%), it is unlikely to meaningfully influence our results. Finally, it is important to note that our patient adherence patterns may not be generalizable to other populations. However, our findings relating clinical characteristics to adherence are more likely to be reflected in other settings.

## Conclusions

This population-based study of premenopausal breast cancer patients found that women with stage I disease, higher comorbidity burden, and/or a history of use of medications for psychiatric disorders were at a higher risk of poorer AET adherence. These at-risk groups of women may benefit most from interventions aimed at encouraging long-term adherence.

### Supplementary Information

**Additional file 1. Supplementary Table S1.** Anatomical Therapeutic Chemical (ATC) classification codes for selected non-cancer chronic medications; **Supplementary Figure S1.** Flow chart for study inclusion; **Supplementary Figure S2** A-K. Directed Acyclic Graphs; **Supplementary Table S2.** Bayesian Information Criteria (BIC) values for number of groups; **Supplementary Table S3**. Varying the order of the polynomials for the best performing trajectory model (3-group model); **Supplementary Figure S3** A-C. Distributions of the probability of group-membership by assigned trajectory group; **Supplementary Figure S4** A-C. Clustered spaghetti plots for the selected three-group trajectory model; **Supplementary Figure S5**. Results from sensitivity analysis changing the definition of prior medication use to a period further from the time of breast cancer diagnosis.

## Data Availability

In accordance with Danish data privacy laws, all sensitive health data used in this study were compiled and analyzed within the secure servers of Statistics Denmark and are not accessible. The detailed study protocol can be made available upon request to the corresponding author.

## Abbreviations

AET: Adjuvant endocrine therapy
ER+: Estrogen-receptor positive
TAM: Tamoxifen
AI: Aromatase inhibitor
DBCG: Danish Breast Cancer Group
HR: Hazard ratio
CI: Confidence intervals
PDC: Proportion of days covered
ProBe CaRe: The Predictors of Breast Cancer Recurrence cohort
CCI: Charlson Comorbidity Index
ATC: Anatomic therapeutic chemical
HER2: Human epidermal growth factor receptor 2
DAGs: Directed acyclic graphs
OR: Odds ratio
