# Supplementary material for: Clinical factors associated with patterns of endocrine therapy adherence in premenopausal breast cancer patients
Source: Breast Cancer Res. 2024 Apr 8;26:59. doi: 10.1186/s13058-024-01819-4 (PMC11003111; doi:10.1186/s13058-024-01819-4)
Supplement: Supplementary file 1 — Additional file 1. Supplementary Table S1. Anatomical Therapeutic Chemical (ATC) classification codes for selected non-cancer chronic medications; Supplementary Figure S1. Flow chart for study inclusion; Supplementary Figure S2 A-K. Directed Acyclic Graphs; Supplementary Table S2. Bayesian Information Criteria (BIC) values for number of groups; Supplementary Table S3. Varying the order of the polynomials for the best performing trajectory model (3-group model); Supplementary Figure S3 A-C. Distributions of the probability of group-membership by assigned trajectory group; Supplementary Figure S4 A-C. Clustered spaghetti plots for the selected three-group trajectory model; Supplementary Figure S5. Results from sensitivity analysis changing the definition of prior medication use to a period further from the time of breast cancer diagnosis. [file 13058_2024_1819_MOESM1_ESM.docx]

**Supplementary Materials:** Clinical factors and patterns of endocrine therapy adherence in premenopausal breast cancer

**Authors:** Kirsten M. Woolpert, MPH (1), Julie A. Schmidt, PhD (1), Thomas P. Ahern, PhD (2), Cathrine F. Hjorth, PhD (1), Dóra K. Farkas, MSc (1), Bent Ejlertsen, MD (3, 4), Lindsay J. Collin, PhD (5), Timothy L. Lash, DSc (6), Deirdre P. Cronin-Fenton, PhD (1)

**Contents**:

[**Supplementary Methods** 2](#_Toc152587580)

[**Supplementary Table S1.** Anatomical Therapeutic Chemical (ATC) classification codes for selected non-cancer chronic medications 2](#_Toc152587581)

[**Supplementary Figure S1.** Flow chart for study inclusion 3](#_Toc152587582)

[**Detailed criteria for group-based trajectory selection** 4](#_Toc152587583)

[**Supplementary Figure S2 A-K.** Directed Acyclic Graphs 7](#_Toc152587584)

[**Supplementary Results** 8](#_Toc152587585)

[**Detailed results from group-based trajectory modeling** 8](#_Toc152587586)

[**Supplementary Table S2**. Bayesian Information Criteria (BIC) values for number of groups 9](#_Toc152587587)

[**Supplementary Table S3.** Varying the order of the polynomials for the best performing trajectory model (3-group model). 10](#_Toc152587588)

[**Supplementary Figure S3 A-C**. Distributions of the probability of group-membership by assigned trajectory group. 11](#_Toc152587589)

[**Supplementary Figure S4 A-C.** Clustered spaghetti plots for the selected three-group trajectory model. 12](#_Toc152587590)

[**Supplementary Figure S5.** Associations of prior non-cancer chronic medication use with adherence to adjuvant endocrine therapy among 4,353 premenopausal breast cancer patients: Results from sensitivity analysis changing the definition of prior medication use to a period further from the time of breast cancer diagnosis. 13](#_Toc152587591)

[**References** 14](#_Toc152587592)

# Supplementary Methods

## **Supplementary Table S1.** Anatomical Therapeutic Chemical (ATC) classification codes for selected non-cancer chronic medications

| Substance Name | ATC code prefix |
| --- | --- |
| Psychoanaleptics | N06 |
| Psycholeptics | N05 |
| Thyroid medications | H03 |
| Systemic hormonal contraceptives | G03A |
| Analgesics | N02 |
| Diuretics or antihypertensives | C02 or C03 |
| Obstructive airway or systemic antihistamines | R03 or R06 |


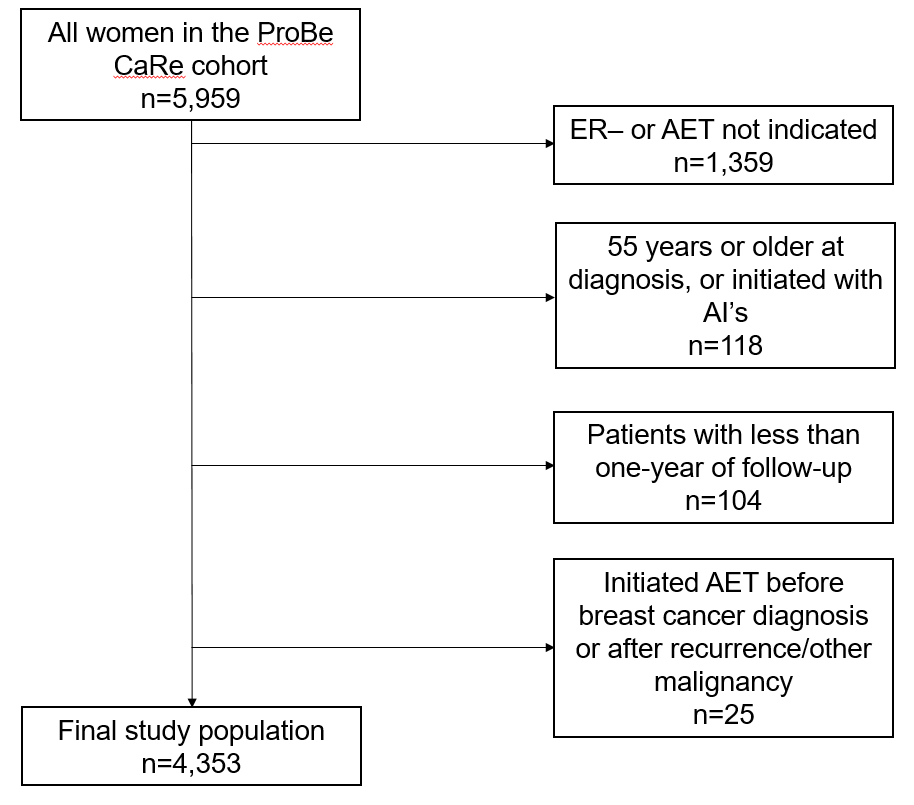


## **Supplementary Figure S1.** Flow chart for study inclusion

Abbreviations: ProBe CaRe, Predictors of Breast Cancer Recurrence; ER-, estrogen receptor negative; AET, adjuvant endocrine therapy; AIs, aromatase inhibitors

## **Detailed criteria for group-based trajectory selection**

#### Step one: Selecting the appropriate number of groups

We started with quadratic (second order) polynomials for the first step of the model selection process. The reason was that we had less than 12 time points, and higher polynomials may lead to overfitting.^1,2^ In this step, we varied the number of groups from two to seven, recording both the Bayesian Information Criteria (BIC) and the Bayes Factor (2*(BICi+1-BICi)).^3^ As suggested in previous literature, a decreasing BIC indicates better model fit.^1,3,4^ At this step in the selection process, we also wanted to consider the clinical relevance of selected groups, and avoid small groups. For example, if two separate groups emerged, but appeared to have similar clinical relevance (*i.e.*, the patterns in the groups were similar), we would opt for the model with fewer groups.

#### Step two: Determining functional forms of each group in the final selected model

We selected the best-performing model from Step One for further analysis. To select the appropriate order of polynomials for each group in the selected model(s), the following criteria were considered:^1,3,5^

1. The largest BIC value.
2. Each group should be populated by more than 5% of patients.
3. No model overfitting, as assessed visually (*i.e.*, a horizontal line should be fit with a constant polynomial, and not a higher order polynomial).
4. A coefficient for the highest polynomial parameter that was different from 0 (*i.e.*, a p<0.05 for the highest order polynomial).
5. Consistent confidence intervals over time for each group.

Step three: Post-selection assessment of the selected model(s)

1. The distribution of the maximum posterior probability of group membership, using box-and-whisker plots. An average >0.7 is often considered to indicate a good model fit, but we also inspected any outliers in the distribution. Due to Danish data protection laws, these box-and-whisker plots could not be published but were visually assessed within Statistics Denmark’s servers.
2. A comparison of the proportion of the population estimated to belong to each group with the proportion assigned by the model to each group. Smaller differences are desired.
3. Odds of correct classification, where a higher value (greater than 5) indicates a better model fit. The odds of correct classification is a measure the ratio of correct classifications within each subgroup, calculated using the maximum probability classification rule and the estimated class membership proportions.^6^
4. Relative entropy, which quantifies the level of classification accuracy when assigning participants to a trajectory according to their posterior probabilities.^6^ It ranges from 0 (highly uncertain) to 1 (highly certain).
5. Individual level spaghetti plots, which were visually inspected to ensure that individual-level trends in adherence patterns matched well to the assigned adherence group. These were constructed by randomly selecting 200 individuals from each of the three groups and plotting their longitudinal PDC values.
6. Clustered spaghetti plots (i.e., tagliatelle plots), which were created for publication purposes. These were constructed by randomly selecting 200 individuals from each of the three groups, then assigning each individual to one of 40 clusters. Those clusters were then plotted for their average longitudinal PDC values.


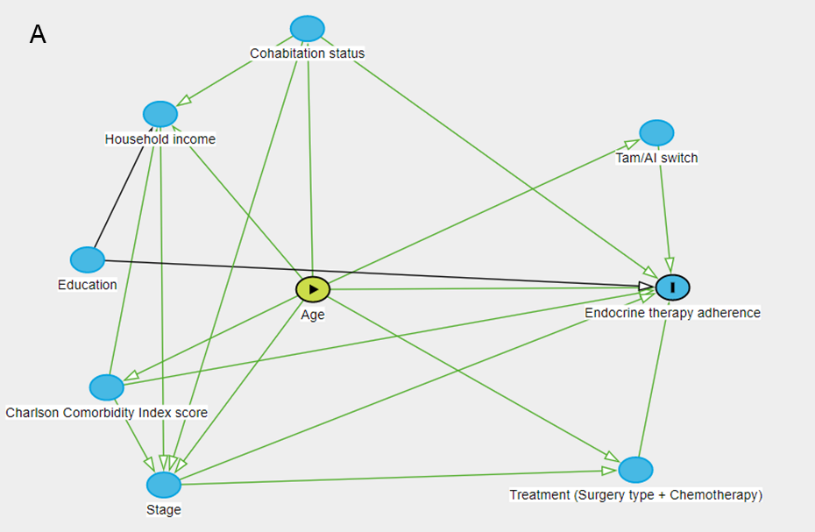

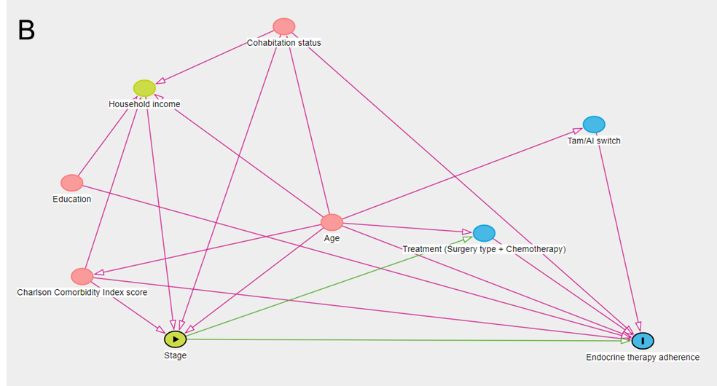


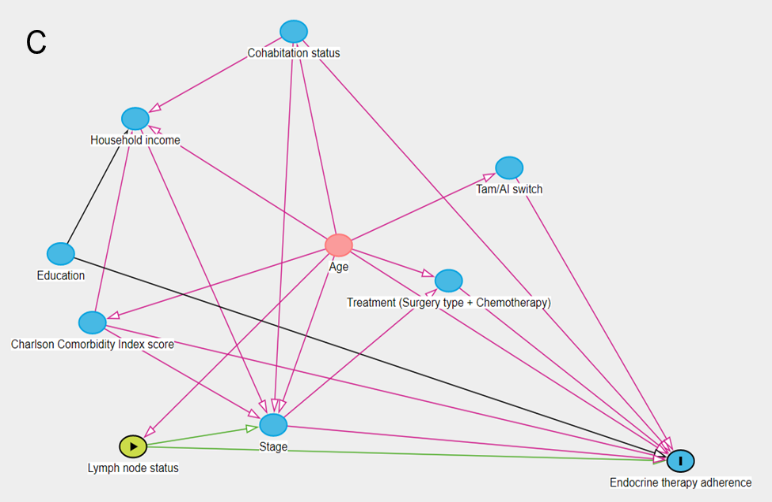

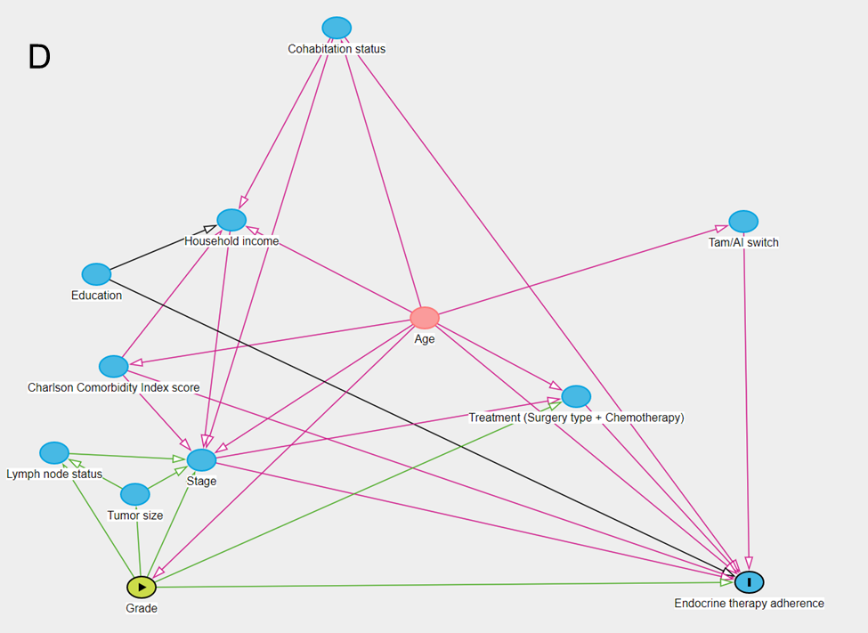


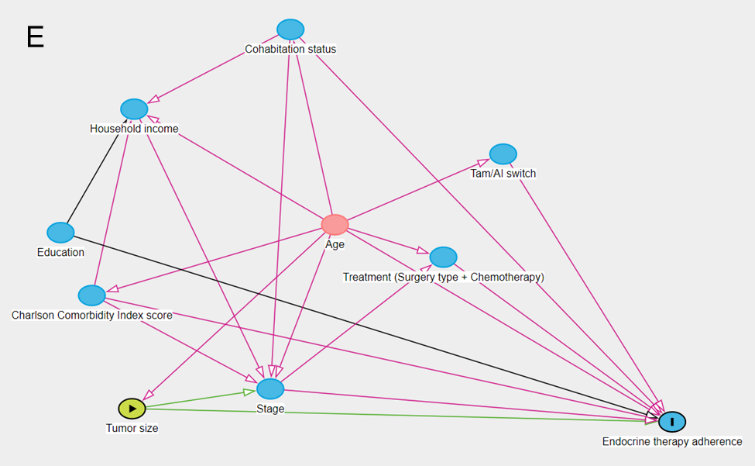

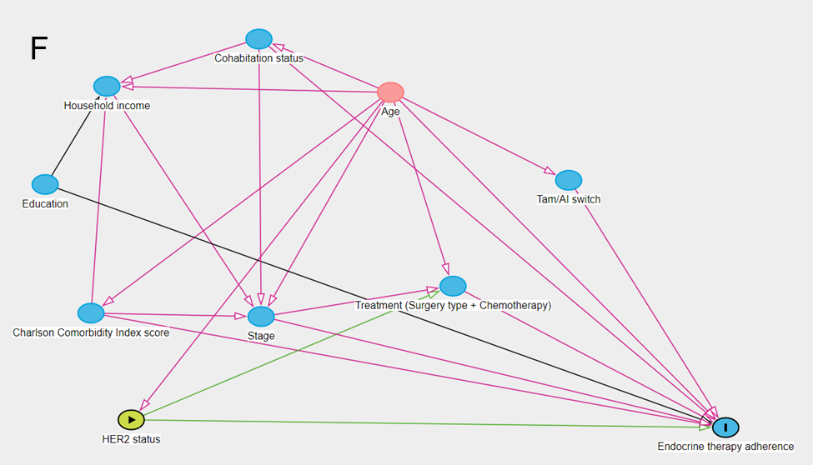


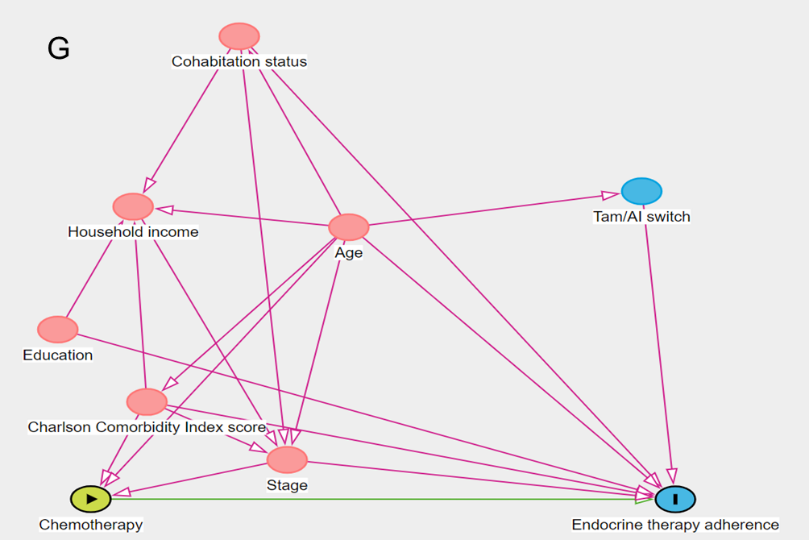

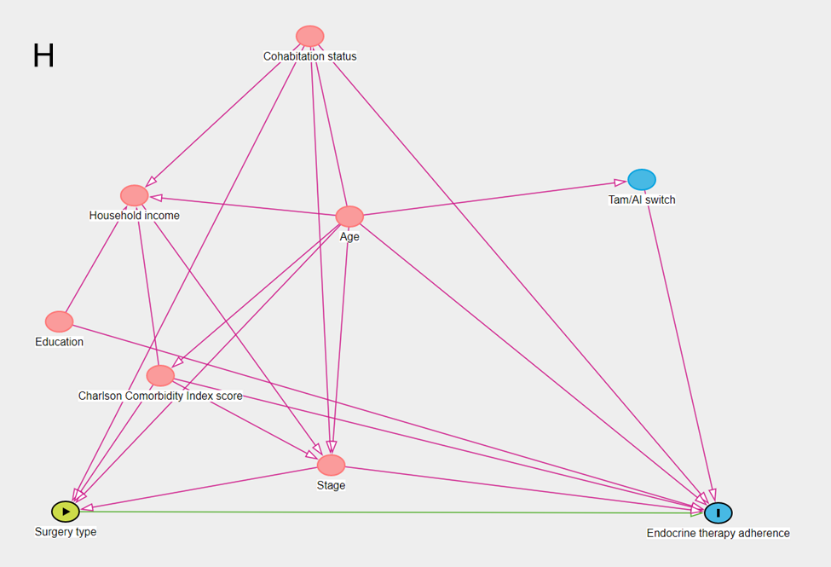

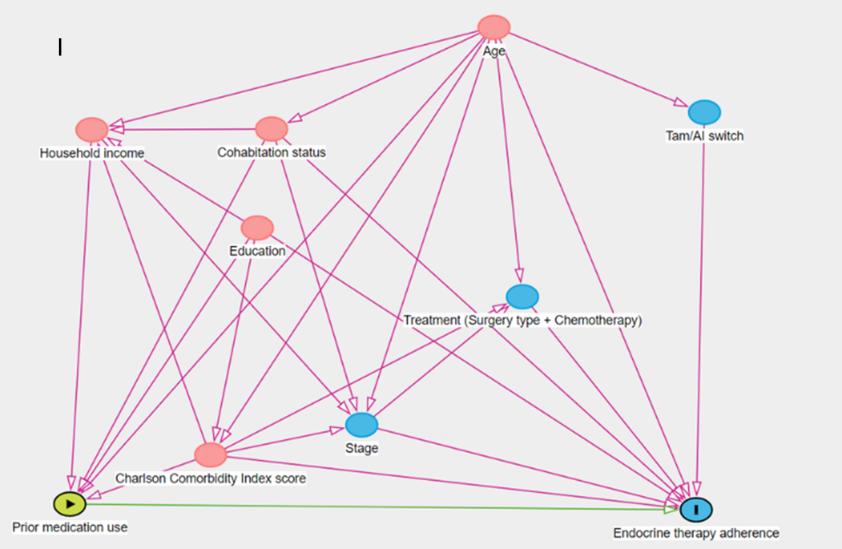

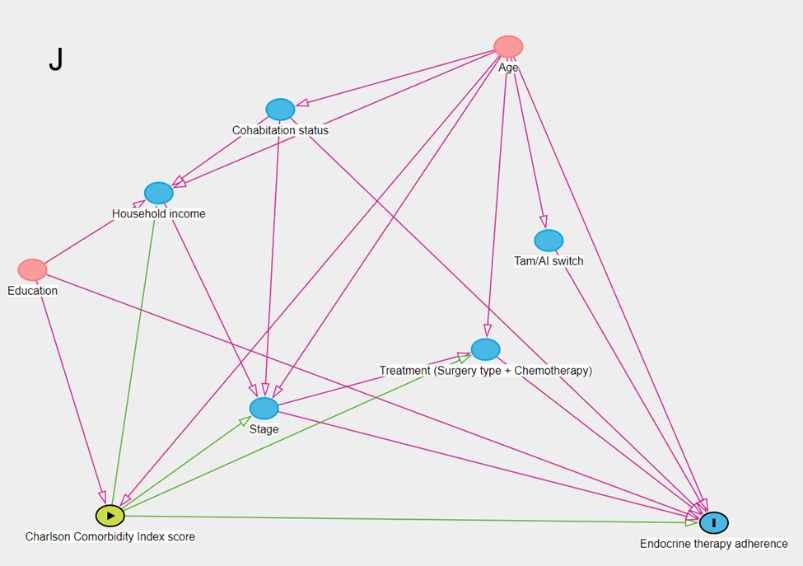


**Supplementary Figure S2 A-K.** Directed Acyclic Graphs for the associations between clinical characteristics, baseline comorbidities, and prior non-cancer chronic medication use and subsequent adjuvant endocrine therapy (AET) adherence in a cohort of premenopausal breast cancer patients.

A. Association between age and AET adherence; B. Association between cancer stage at diagnosis and AET adherence; C. Association between lymph node status and AET adherence; D. Association between histological grade and AET adherence; E. Association between tumor size at diagnosis and AET adherence; F. Association between HER2 status and AET adherence; G. Association between chemotherapy and AET adherence; H. Association between surgery type/radiation and AET adherence; I. Association between prior medication use and AET adherence; J. Association between Charlson Comorbidity Index score and AET adherence;

# Supplementary Results

## **Detailed results from group-based trajectory modeling**

#### Step one: Selecting the appropriate number of groups

We selected the three-group model as our final model for further analysis. Adding additional groups decreased the Bayes Factor, resulting in an additional improvement in the model (Supplementary Table S1). However, the trajectories between the three- and four- group models were determined to be clinically similar by our team.

Step two: Determining functional forms of each group in the final selected model

In the final selected three-group model, we reran the models with every possible combination of 0 to 2^nd^ order polynomial, e.g., 3^3^=27 possible combinations (Supplementary Table S2). The lowest BIC value was observed in the model with all three groups modeled with 2^nd^ order polynomials. However, upon visual inspection, it was determined that modeling our high adherers group with this level of flexibility may lead to overfitting, and rather that our high adherers should be modeled with a 0-order polynomial (a constant). Keeping this in mind, our fifth highest BIC value, in our 2 2 0 group (i.e., 2^nd^ order for slow and rapid decliners, constant for high adherers) was our final selected model.

Step three: Post-selection assessment of the selected model(s)

We further assessed the performance of our three-group model with declining adherence modeled with second order polynomials, and high adherence modeled with a constant polynomial. Other considered criteria, such as odds of correct classification, relative entropy, average posterior probabilities, and probabilities of group membership are shown in Supplementary Table 2S. Visual assessment of box-and-whisker plots within the Statistics Denmark servers showed a high probability of group membership for most of the study cohort, with few outliers (Supplementary Figure S3). The patterns that emerged both in the tagliatelle plots (Supplementary Figure S4 A-C) and individual-level spaghetti plots (data not shown) were also consistent with the overall group patterns.

Supplementary Table S2. Bayesian Information Criteria (BIC) values for number of groups

| Number of groups | Order of the polynomials | BIC | Bayes Factor^1^ |
| --- | --- | --- | --- |
| 2 | 2 | -16508 | . |
| 3 | 2 | -14411 | 4195 |
| 4 | 2 | -14045 | 732 |
| 5 | 2 | -13469 | 1153 |
| 6 | 2 | -13382 | 174 |
| 7 | 2 | -13144 | 475 |

1. Bayes Factor calculated as (2*(BICi+1-BICi)).

## **Supplementary Table S3.** Varying the order of the polynomials for the best performing trajectory model (3-group model).

| Order of the polynomials | BIC value | Percent of participants (rapid decliners) | Percent of participants (slow decliners) | Percent of participants (high adherers) | Average posterior probability (rapid decliners) | Average posterior probability (slow decliners) | Average posterior probability (high adherers) | Probability of membership (rapid decliners) | Probability of membership (slow decliners) | Probability of membership (high adherers) | Odds of correct classification (rapid decliners) | Odds of correct classification (slow decliners) | Odds of correct classification (high adherers) | Relative entropy |
| --- | --- | --- | --- | --- | --- | --- | --- | --- | --- | --- | --- | --- | --- | --- |
| 0 0 0 | -16958 | 6.3 | 36.9 | 56.8 | 0.93 | 0.97 | 0.96 | 0.06 | 0.39 | 0.55 | 197 | 58 | 19 | .90 |
| 1 0 0 | -15307 | 8.3 | 35.0 | 56.7 | 0.96 | 0.97 | 0.96 | 0.08 | 0.36 | 0.55 | 253 | 56 | 21 | .91 |
| 2 0 0 | -15278 | 8.5 | 34.8 | 56.6 | 0.95 | 0.97 | 0.96 | 0.09 | 0.36 | 0.55 | 219 | 58 | 20 | .91 |
| 0 1 0 | -16041 | 4.2 | 38.8 | 56.9 | 0.93 | 0.97 | 0.96 | 0.04 | 0.40 | 0.56 | 287 | 47 | 21 | .91 |
| 1 1 0 | -14635 | 7.0 | 36.5 | 56.6 | 0.96 | 0.97 | 0.96 | 0.07 | 0.38 | 0.55 | 322 | 59 | 20 | .91 |
| 2 1 0 | -14592 | 7.1 | 36.4 | 56.5 | 0.96 | 0.97 | 0.96 | 0.07 | 0.38 | 0.55 | 333 | 61 | 19 | .91 |
| 0 2 0 | -15278 | 34.8 | 8.5 | 56.6 | 0.97 | 0.95 | 0.96 | 0.36 | 0.09 | 0.55 | 58 | 219 | 20 | .91 |
| 1 2 0 | -14592 | 36.4 | 7.1 | 56.5 | 0.97 | 0.96 | 0.96 | 0.38 | 0.07 | 0.55 | 61 | 333 | 19 | .91 |
| 2 2 0^1^ | **-14572** | **6.9** | **36.5** | **56.6** | **0.97** | **0.97** | **0.96** | **0.07** | **0.38** | **0.55** | **395** | **57** | **19** | **.91** |
| 0 0 1 | -16713 | 6.2 | 35.4 | 58.4 | 0.92 | 0.96 | 0.97 | 0.06 | 0.36 | 0.58 | 178 | 46 | 28 | .91 |
| 1 0 1 | -15064 | 8.3 | 33.4 | 58.4 | 0.96 | 0.96 | 0.97 | 0.08 | 0.34 | 0.58 | 250 | 50 | 28 | .92 |
| 2 0 1 | -15360 | 8.6 | 39.1 | 52.3 | 0.96 | 0.99 | 0.97 | 0.09 | 0.40 | 0.51 | 244 | 129 | 35 | .94 |
| 0 1 1 | -15898 | 4.3 | 39.1 | 56.6 | 0.92 | 0.98 | 0.97 | 0.04 | 0.40 | 0.56 | 262 | 62 | 29 | .93 |
| 1 1 1 | -14486 | 7.0 | 36.7 | 56.4 | 0.96 | 0.98 | 0.97 | 0.07 | 0.38 | 0.55 | 329 | 66 | 29 | .93 |
| 2 1 1 | -14678 | 7.4 | 40.4 | 52.3 | 0.96 | 0.99 | 0.97 | 0.07 | 0.42 | 0.51 | 313 | 171 | 31 | .94 |
| 0 2 1 | -15037 | 33.3 | 8.3 | 58.4 | 0.96 | 0.96 | 0.97 | 0.34 | 0.09 | 0.58 | 50 | 267 | 27 | .92 |
| 1 2 1 | -14452 | 7.0 | 36.4 | 56.6 | 0.95 | 0.98 | 0.97 | 0.07 | 0.37 | 0.56 | 274 | 66 | 29 | .93 |
| 2 2 1 | -14417 | 6.9 | 36.6 | 56.4 | 0.96 | 0.97 | 0.97 | 0.07 | 0.37 | 0.55 | 356 | 63 | 29 | .93 |
| 0 0 2 | -17031 | 6.5 | 40.5 | 53.0 | 0.94 | 0.99 | 0.97 | 0.07 | 0.42 | 0.51 | 239 | 105 | 30 | .94 |
| 1 0 2 | -15398 | 8.5 | 39.2 | 52.3 | 0.96 | 0.99 | 0.97 | 0.09 | 0.40 | 0.51 | 240 | 137 | 36 | .93 |
| 2 0 2 | -15364 | 8.6 | 39.1 | 52.3 | 0.96 | 0.99 | 0.97 | 0.09 | 0.40 | 0.51 | 244 | 129 | 35 | .94 |
| 0 1 2 | -16114 | 5.0 | 41.8 | 53.2 | 0.92 | 0.99 | 0.97 | 0.05 | 0.43 | 0.52 | 238 | 154 | 30 | .95 |
| 1 1 2 | -14728 | 7.5 | 40.2 | 52.3 | 0.95 | 0.99 | 0.97 | 0.08 | 0.42 | 0.51 | 235 | 154 | 32 | .94 |
| 2 1 2 | -14682 | 7.4 | 40.4 | 52.3 | 0.96 | 0.99 | 0.97 | 0.07 | 0.42 | 0.51 | 313 | 171 | 31 | .94 |
| 0 2 2 | -15822 | 3.6 | 37.7 | 58.6 | 0.94 | 0.97 | 0.97 | 0.04 | 0.39 | 0.58 | 411 | 55 | 24 | .92 |
| 1 2 2 | -14668 | 7.4 | 39.4 | 53.2 | 0.95 | 0.99 | 0.97 | 0.07 | 0.41 | 0.52 | 232 | 138 | 29 | .94 |
| 2 2 2 | -14411 | 6.8 | 35.7 | 57.5 | 0.97 | 0.97 | 0.97 | 0.07 | 0.37 | 0.56 | 383 | 56 | 24 | .92 |

Abbreviations: BIC, Bayesian Information Criteria. (1) This row represents the final selected model

1. High adherers (n=2,465)


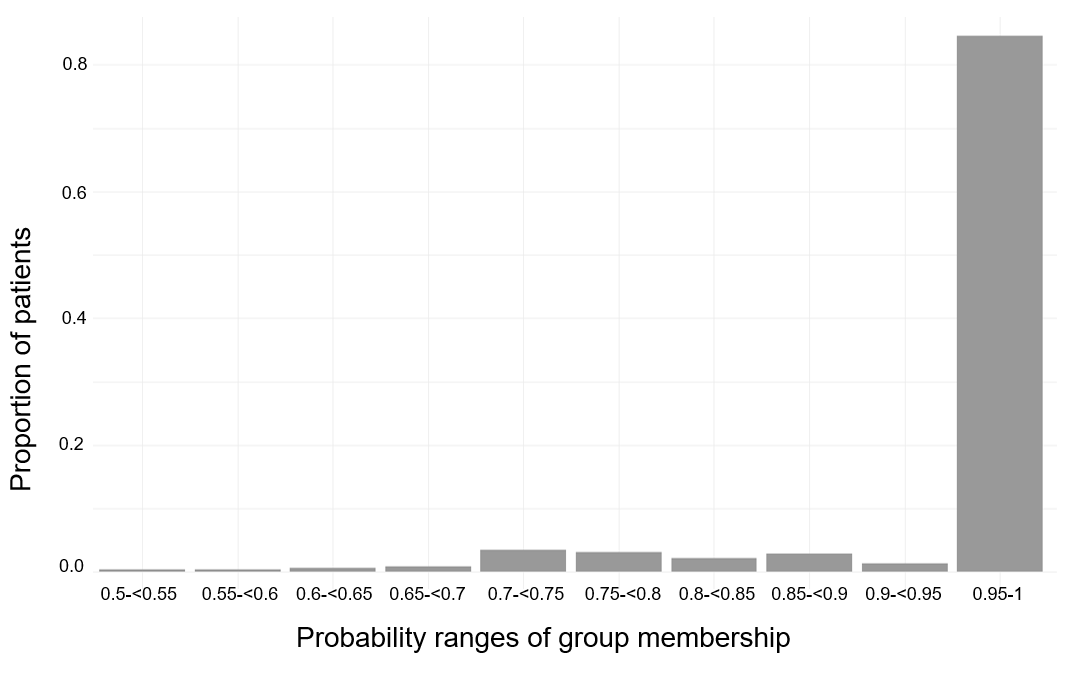


1. Slow decliners (n=1,587)


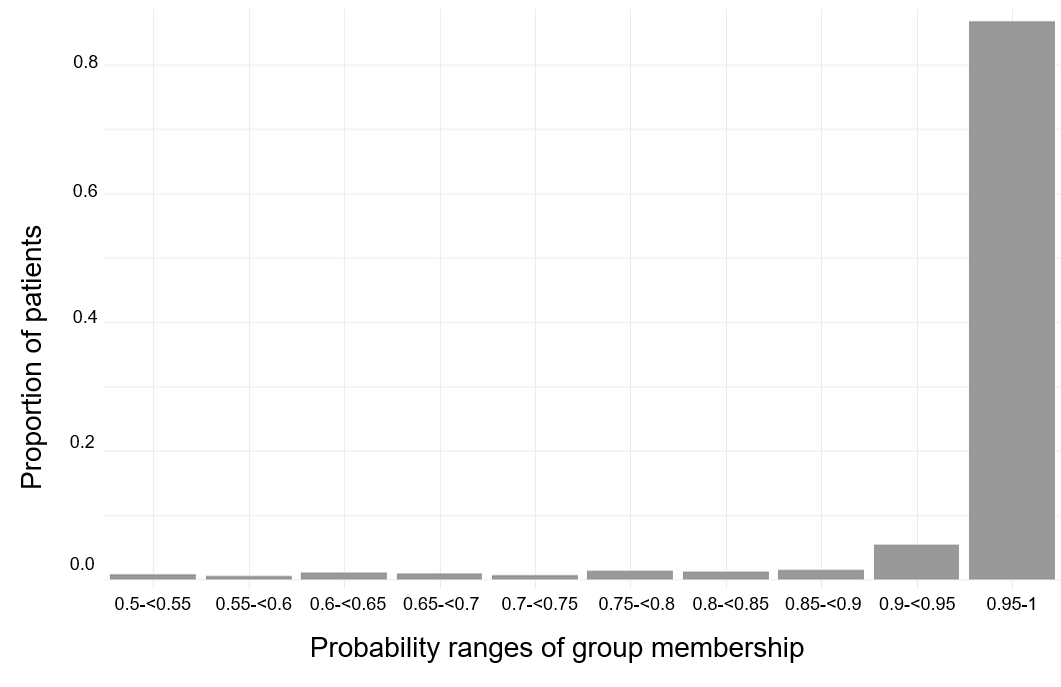


1. Rapid decliners^a^ (n=301)


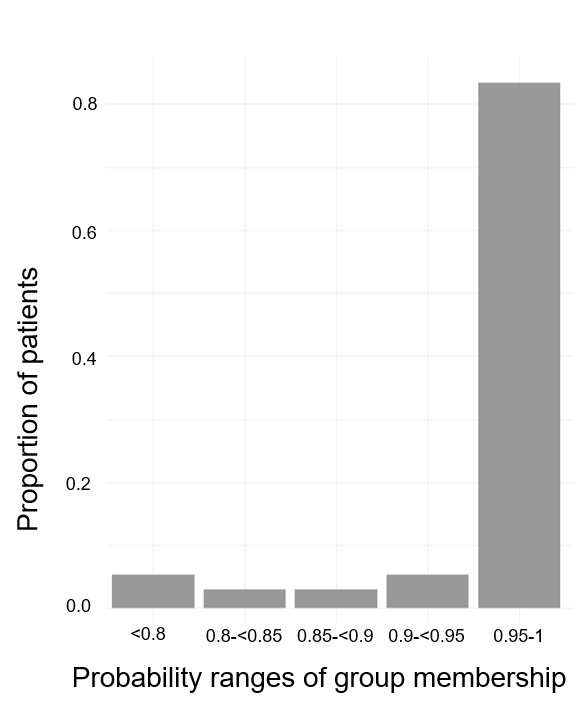


## **Supplementary Figure S3 A-C**. Distributions of the probability of group-membership by assigned trajectory group.

1. Groups with less than five individuals were combined in this plot in accordance with Danish privacy laws into a <0.8 subgroup.
2. High adherers B. Slow decliners


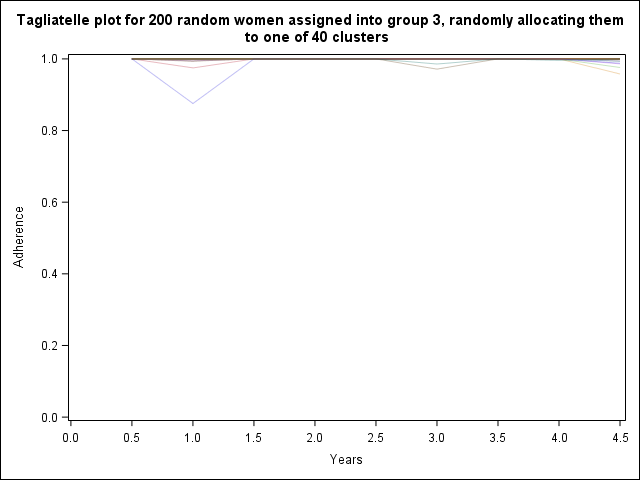

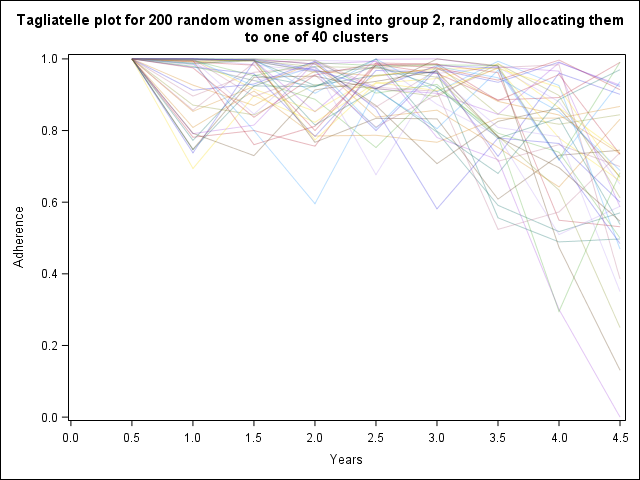


C. Rapid decliners


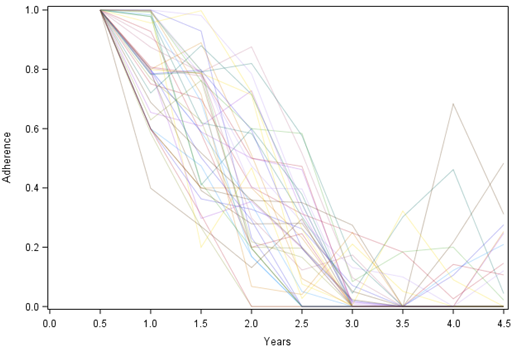


Supplementary Figure S4 A-C. Clustered spaghetti plots for the selected three-group trajectory model.

Plots were constructed by randomly selecting 200 individuals within each group, then randomly allocating them to one of 40 clusters. We took the average proportion of days covered (PDC) at each time point within each of these clusters and plotted the 40 individual trajectories for each group.


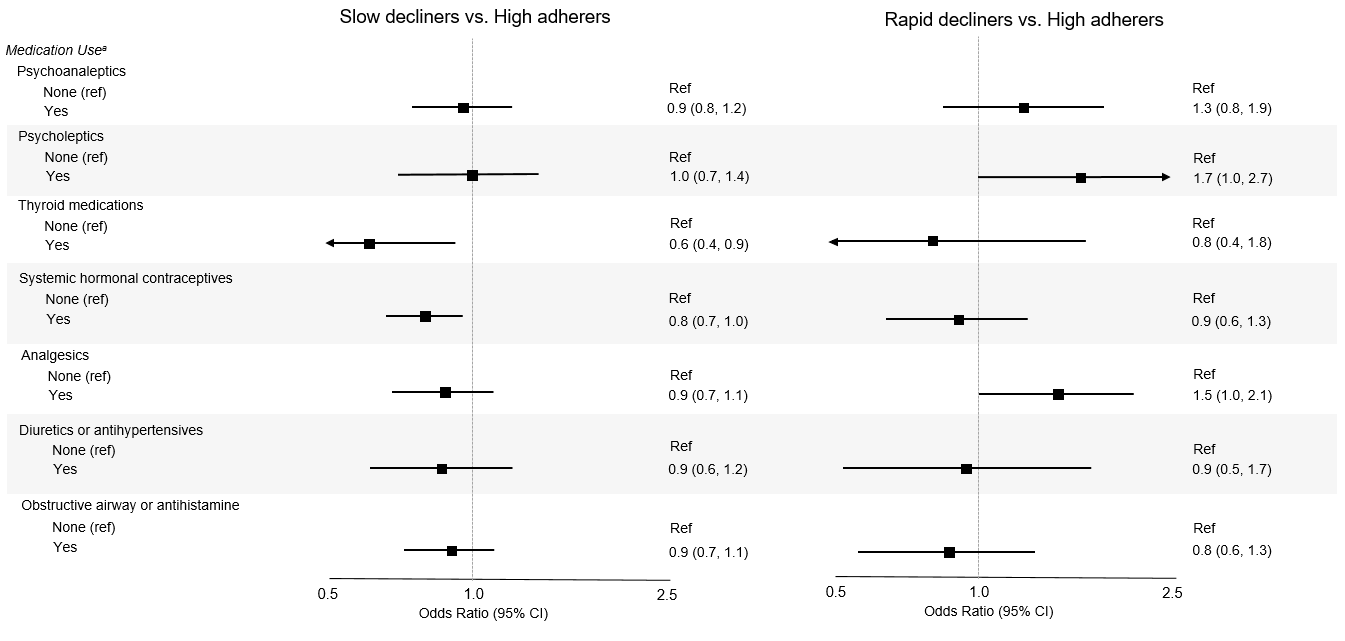


Supplementary Figure S5. Associations of prior non-cancer chronic medication use with adherence to adjuvant endocrine therapy among 4,353 premenopausal breast cancer patients: Results from sensitivity analysis changing the definition of prior medication use to a period further from the time of breast cancer diagnosis.

Abbreviations: OR, odds ratio; CI, confidence interval.

^a^Associations between prior medication use (and all selected medications) and adherence adjusted for age, Charlson Comorbidity Index score, household income, cohabitation status, and education level.

Period for exposure to prior non-cancer chronic medication use defined as one to three years before diagnosis in this sensitivity analysis.

# References

1. Hickson RP, Annis IE, Killeya-Jones LA, Fang G. Opening the black box of the group-based trajectory modeling process to analyze medication adherence patterns: An example using real-world statin adherence data. Pharmacoepidemiol Drug Saf. 2020 Mar;29(3):357–62.

2. Librero J, Sanfélix-Gimeno G, Peiró S. Medication Adherence Patterns after Hospitalization for Coronary Heart Disease. A Population-Based Study Using Electronic Records and Group-Based Trajectory Models. PLOS ONE. 2016 Aug 23;11(8):e0161381.

3. Nagin DS. Group-Based Trajectory Modeling: An Overview. ANM. 2014;65(2–3):205–10.

4. Franklin JM, Shrank WH, Pakes J, Sanfélix-Gimeno G, Matlin OS, Brennan TA, et al. Group-based Trajectory Models: A New Approach to Classifying and Predicting Long-Term Medication Adherence. Medical Care. 2013;51(9):789–96.

5. Nagin DS, Odgers CL. Group-Based Trajectory Modeling (Nearly) Two Decades Later. J Quant Criminol. 2010;26(4):445–53.

6. Lennon H, Kelly S, Sperrin M, Buchan I, Cross AJ, Leitzmann M, et al. Framework to construct and interpret latent class trajectory modelling. BMJ Open. 2018 Jul 7;8(7):e020683.
